# Production and Characterization of a Nitrilase from *Pseudomonas aeruginosa* RZ44 and its Potential for Nitrile Biotransformation

**DOI:** 10.15171/ijb.1179

**Published:** 2016-09

**Authors:** Arastoo Badoei-Dalfard, Narjes Ramezani-pour, Zahra Karami

**Affiliations:** Department of Biology, Faculty of Sciences, Shahid Bahonar University of Kerman, Kerman, Iran

**Keywords:** Nitrilase, Nitrile, Nitrile-degrading bacteria, Production, *Pseudomonas aeruginosa*, Sewage

## Abstract

**Background:**

The conversion of nitriles into amides or carboxylic acids by nitrilase has taken its application into consideration, as the scope of its applications has recently been extended.

**Objectives:**

In this study, *P. aeruginosa* RZ44 was isolated from sewage in the Kerman which has Nitrile-degradation activity. In order to improve the nitrilase production, several optimization were done on environmental condition. Nitrilase activity was characterized against different pHs, temperatures, ions, and substrates.

**Materials and Methods:**

Enzyme activity was evaluated by determining the production of ammonia following to the modification of the phenol/hypochlorite method. Different factors that affect production of the enzyme by *P. aeruginosa* RZ44 were optimized and evaluated in the culture mediums.

**Results:**

The results showed that degradation of the acetonitrile by *P. aeruginosa* RZ44 increased the pH of the growth medium from the initial pH 7.0 to 9.37. Optimizing the medium for *P. aeruginosa* RZ44, it was found that glucose and starch (5 g.L^-1^) have strongly supported nitrilase production, compared to the control. As well, urea (5 g.L^-1^) and yeast extract (15 g.L^-1^) have favored an increased biomass and nitrilase production, as the nitrogen sources. These results show that nitrilase production increases in the pH range 5.0 to 7.0 and then start decreasing. Addition of the Mg^2+^, Fe^2+^ and Na^+^ has supported the biomass and nitrilase production. Co^2+^, Mn^2+^ and Cu^2+^ were confirmed to inhibit cell growth and enzyme production. Enzyme characterization results show that, *P. aeruginosa* RZ44 nitrilase exhibits comparatively high activity and stability at pH 7.0 and 40°C. Nitrilase was completely inhibited by CoCl_2_ and CaCl_2_, whereas, the inhibition in the presence of MnSO_4_ and CuSO4 was about 60%. Time course analysis of the nitrile conversion by the resting * P. aeruginosa * RZ44 cells showed that nitrile substrates (i.e. acetonitrile) was hydrolyzed within 8 h.

**Conclusions:**

these results indicate that *P. aeruginosa* RZ44 has the potential to be applied in the biotransformation of nitrile compounds.

## 1. Background

Nitriles are broadly used in the organic synthesis of‏ the amides, carboxylic acids, and their derivatives (1-6). In addition, these compounds are presumed to have‏ an abundant impact in the chemical industrial procedures, where they are primarily used for the production of the numerous fine chemicals (1), in the ecological sciences, as the carcinogenic compounds, neurotoxic, as well as ecological contaminants (7). Chemical hydrolysis of the nitriles often includes fairly severe situations that impede the use of nitriles carrying sensitive functionalities. Furthermore, the undesirable byproducts, including large amount of salt, are regularly produce (8). Biotransformation of the nitriles by nitrile hydrolyzing enzymes (1,9), overcomes these problems and may deal with an extra benefit of the stereospecificity (10-13). The use of nitrile-degrading enzymes has expanded its abundant interest among scientists for the conversion of the nitriles to the valuable chemical products (14), or reclamation of the nitrile polluted soil/water (15,16). To these interest it could be added the production of the commercially feasible compounds, such as acrylamide (17), antibiotics, anti-inflammatory agents (18), as well as herbicides (10,19). Microbial degradation has been investigated as an effective approach for eliminating the highly poisonous nitriles from the environment. Some of the recently reported nitrle-hydrolyzing bacteria include: *Alcaligenes* sp. (3, 8), *Arthrobacter nitroguajacolicus* (14), *Nocardia globerula* NHN-2 (20), *Serratia marcescens* (21), *Pseudomonas* (4,13,22,23), *Geobacillus pallidus* (24), *Rhodococcus rhodochrous* PA-34 (25), and *Isoptericola variabilis* RGT01 (26). In spite of this notion, there are only few reports about optimization of the different factors for the maximum production of nitrilases.

## 2. Objectives

In this study, *P. aeruginosa* RZ44 was isolated from sewage in the Kerman which has Nitrile-degradation activity. In order to improve the nitrilase production from *Pseudomonas aeruginosa* RZ44, several optimization were done on environmental condition. Nitrilase activity was biochemically characterized against different pHs, temperatures, ions, and substrates.

## 3. Material and Methods

### 3.1. Chemicals

All substrates were procured from Merck (White house Station, New Jersey, United States). The culture media ingredients were from Sigma (St. Louis, MO, SA). All the other chemicals were of analytical grade and purchased from various suppliers.

*3.2. Screening of the Acetonitrile-Degrading Bacteria* Soil and water samples were picked up from the sewage of the Kerman city, Iran. Nitrile-degrading bacteria were screened through application of the enrichment culture and indicator plate methods. Cultures were grown in the mineral salts medium (MM1), which was lacking for carbon and nitrogen source and contained the following components (%): K_2_HPO_4_ 0.68; KH_2_PO_4_ 0.12; MgSO_4_.7H_2_O 0.01; MnSO_4_. 4H_2_O 0.01; CaCl_2_.2H_2_O 0.01; FeSO_4_.7H_2_O 0.01; Na_2_MoO_7_.2H_2_O 0.0006 (27). The pH of the medium was adjusted to 7.0 and autoclaving at 121°C was done for 15 min. The bacteria were propagated using MM1 medium complemented with 1% filtered sterile acetonitrile (0.4 M) as the sole source of nitrogen, carbon, and energy. The flasks were inoculated and incubated at 30°C in an orbital shaker at 168 rpm. Indicator plates were prepared from a MM1 medium with phenol red (0.015% (w/v)) and 1.5% agar, overlaid with 100 μL nitriles.

### 3.3. Identification of the Acetonitrile-Degrading Bacteria

The *P. aeruginosa* RZ44 was identified by using the 16S rRNA gene sequencing method. The DNA of the bacteria was extracted according to the *Sambrook* and *Russell* (28). The 16S ribosomal genes were amplified from the bacterial total genomic DNA by using polymerase chain reaction (PCR). Universal 16S rRNA forward primer (5´-AGTTTGATCCTGGCTCAG-3´) and the reverse primer (5´-GGCACCTTGTTACGACTT-3´) were used for the PCR amplification of the 16S rRNA gene (29). Each 200 μL microtube contained 2 μL of the purified extracted DNA; 1.5 μL of dNTP at 2.5 mM; 2.5 μL of 10X buffer; 0.2 μL of 2.5 unit *Taq* DNA polymerase; 16.8 μL of the sterile water and 1 μL of the primers (final concentration: 10 pmol). PCR was performed in a Biorad PCR system thermal cycler with a hot starting of the reaction performed at 95°C for 5 min, followed by 33 cycles of 94°C for 1 min, 54°C for 0.45 min, and 72°C for 1 min, followed by a final extension at 72°C for 10 min. PCR products were electrophoresed on agarose gel (1%), and subsequently the amplified 16S rRNA bands were purified by applying DNA extraction kit (Cinaclone, Iran). DNA sequencing was performed on both strands directly by SEQ-LAB (Germany).

The phylogenetic tree was constructed based on the comparison between 16S rRNA sequences obtained for *P. aeruginosa* RZ44 with those other strains of*Pseudomonas* species that were obtained from GenBank database (http://www.ncbi.nlm.nih.gov). All sequences were aligned applying Clustal Omega (http://www.seqtool.sdsc.edu/CGI/Omega.cgi) and the phylogenetic tree was constructed in MEGA4 (30). The obtained 16S rRNA sequence was deposited in GenBank for isolate *P. aeruginosa* RZ44, with KM229744 accession number.

### 3.4. Nitrilase Purification

The pellet was gathered by centrifugation at 8000 g for 10 min, subsequently washed with 0.05 M sodium phosphate buffer having 1 mM dithiothreitol and 20% (v/v) glycerol (pH 7.5), and re-centrifuged for 20 min at 13000 rpm. The pellet was then suspended in a minimal volume of 0.05 M sodium phosphate buffer (pH 7.5) (19). Cell were suspended and then disrupted by sonication on ice (5 min, 35 kHz, sonicator, hielscher, Germany). The disrupted cells were centrifuged at 13000 rpm for 20 min and supernatant was collected. The supernatant was fractionated stepwise through precipitation with the ammonium sulfate powder at different saturation. The precipitate formed in each step was collected by centrifugation at 12000 rpm for 20 min, dissolved in 1 mL 50 mM potassium phosphate buffer and assayed for enzyme activity. The fraction with the highest nitrilase activity was dialyzed overnight against Tris/HCl buffer (pH 7.5) to remove the remaining salt. The desalted samples were directly loaded onto a Q-sepharose (1.5×24 cm) previously equilibrated with 45 mM potassium phosphate buffer (pH 7.2) and the column was eluted with a linear gradient of 0-1.0 M NaCl at a flow rate of 1 mL.min^-1^. The nitrilase activity in the eluted fractions was determined, and the active fraction from the previous step was further purified by gel filtration using Sephadex G-100 (1×100 cm). The fractions showing nitrilase activity were pooled and used as RZ44 nitrilase for the following studies. The nitrilase purity was checked with SDS-PAGE (10% gel) under reducing conditions.

### 3.5. Acetonitrile-Degrading Enzyme Assay

Enzyme activity was evaluated by determining the production of ammonia following to the modification of the phenol/hypochlorite method (31). The following components were used for ammonia detection. Reagent A contained 0.6 M phenol and 0.001 M sodium nitroprusside. Reagent B contained 0.11 M sodium hypochlorite and 2.1 M sodium hydroxide (19). The standard assay was done in duplicate at 37°C in a reaction containing 180 μL potassium phosphate buffer (0.1 M, pH 7.5), 20 μL enzyme, 100 μL of the 150 mM acetonitrile or 1.2 M acetamide. Samples were incubated at 37°C for 20 min. The reaction was quenched by addition of 100 μL of the assay mixture to 300 μL reagent B followed by rapid addition of 300 μL reagent Awith vigorous mixing and incubation at 45°C for 20 min. The absorbance was then measured in a spectrophotometer (Biochrom WPA Biowave II). One unit of nitrile degrading enzyme activity was defined as the amount of enzyme capable of releasing 1 μmol ammonia.min^-1^ under standard reaction situations (pH 7.5, 45oC, 50 mM acetonitrile as substrate) (19).

### 3.6. Effect of the Culture Conditions on the Enzyme Production

Different factors that affect production of the enzyme by *P. aeruginosa* RZ44 were optimized and evaluated in the culture mediums with a different pH (i. e. 5.0, 6.0, 7.0, and 8.0). Several carbon sources were used including: glucose, mannitol, acetate, citrate, glycerol, sucrose, fructose, starch, and maltose with the same concentration as subsequently were inoculated with the *P. aeruginosa* RZ44. As well, several nitrogen sources including: yeast extract, peptone, urea, sodium nitrate, and Triton X-100, all at the same concentration were also considered and were inoculated with the *P. aeruginosa* RZ44. In addition, several ion sources were also studied, including: ZnSO_4_, CuSO_4_, MnSO_4_, MgSO_4_, FeSO4, CoCl_2_, CaCl_2_, and NaCl with the same amount that was followed by inoculation of the *P. aeruginosa* RZ44 in the medium supplied with the ion source under study (21,32-34).

### 3.7. Enzyme Characterization

#### 3.7.1. Effect of pH and Temperature on Enzyme Activity and Stability

The effect of pH and temperature on nitrilase RZ44 was considered by incubating the enzyme with substrate in buffers of diverse pHs (pHs from 4 to-10) or at diverse temperatures (from 30 to 90oC). The optimum pH for the enzyme was considered by using the sodium acetate (pH 4-6), phosphate (6-8), and Tris-HCl (8-10) buffers. The enzyme was incubated in 1mL of the respective buffer with substrate (10 mM) in a reaction microtube at room temperature (21,35). For determination of the optimum temperature, the enzyme was incubated in 1 mL of potassium phosphate buffer (50 mM, pH 7.0) with substrate (10 mM)in a reaction vial at 30 to 90oC. The reactions stopped after 20 min by addition of 10 μL of 2 M HCl (23).

#### 3.7.2. Effect of Metal Ions and other Chemicals

To study the effect of metal ions on the enzyme activity, the enzyme was assessed under standard condition and in the presence of different metal ions(CaCl_2_, CoCl_2_, ZnCl_2_, FeSO_4_, CuSO_4_, MgSO_4_, MnSO_4_, ZnSO_4_, DTT, EDTA, and HgCl_2_) supplemented with potassium phosphate buffer (0.1 M, pH 7.5) and chemicals with a final concentration of 1 mM (23,36). Acetonitrile (10 mM) was used as the substrate and the enzyme activity was measured as described above. The measured activities were compared with the activity of the enzyme under the same condition in a mixture without addition of any ion.

#### 3.7.3. Determination of Kinetic Parameters

The specific activity of the crude enzyme toward acetonitrile and acetamide was investigated by quantification of the amount of ammonia released during hydrolysis. The standard reaction mixture (400 μL) was composed of 50 mM potassium phosphate buffer (pH 7.0), 10 mM of substrate, 10 μL of methanol, and an appropriate amount of the enzyme. Initial velocities at pH 7.0 were determined for enzyme by calculating the initial rate of substrate hydrolysis in the range of 1 mM to 60 mM. *K*_m_ and *V*_max_ values were determined from Lineweaver-Burk plots using standard linear regression Methods.

#### 3.7.4. The Substrate Activity Profile of RZ44 Nitrilase

The RZ44 nitrilase was incubated for 30 min in 300 μL of the sodium phosphate buffer (pH 7.0, 50 mM) plus 5% (v/v) methanol, and 10 mM concentration of the respective nitriles. A range of nitrile compounds were used as substrates including saturated nitrileamide (acetonitrile and acetamide), unsaturated nitrileamide (acrylonitrile and acrylamide), cyclic nitrileamide (3-cyanopyridine), aromatic nitrile (benzonitrile, benzoamide, 2-amino benzonitrile) and different amide substrates (23). The reaction was stopped by the addition of 10 μL of HCl (2.0 M) and the precipitated protein was removed by centrifugation in a Helmer centrifuge (12,000 ×*g*, 5 min). The amount of ammonia in the supernatant was measured as described above.

#### 3.7.5. Biotransformation

*P. aeruginosa* RZ44 cells were cultivated in 250-mL Erlenmeyer flasks containing 50 mL of the optimized medium (glucose 10 g.L^-1^, yeast extract 5 g.L^-1^, KH_2_PO_4_ 2 g.L^-1^, NaCl 1 g.L^-1^, MgSO_4_ 0.2 g.L^-1^, nitrile 20 mM, pH 7.0) at 30°C in rotating shaker (168 rpm). After 48 h, cells were centrifuged and suspended in 100 mM phosphate buffer (pH 7.5). The reaction, consisting of the nitrile (at final concentrations of 20 mM) and 20 mg (wet weight) of resting cells in 10 mL phosphate buffer (pH 6.5, 100 mM), was carried out at 30°C and 168 rpm. Samples (100 μL each) from the reaction mixture were taken at regular intervals, and cells were removed by centrifugation (21, 37, 38). The amount of the produce ammonia was measured as described above and an analytical method was done as follows. The amount of nitrile, amide, and acid were also determined by gas chromatography (GC, CP-3800, Varian, United States) using CP8740 column (30 M, 0.32 mm, 0.1 μm) equipped with a flame ionizationdetector (FID). The column, injector and detector temperatures were set at 180, 230 and 250oC, respectively. The flow of helium was maintained at 30 mL.min^-1^. Five μL of the reaction mixture was injected and the residual substrate concentration was calculated against the standard curve (32, 38, 39).

## 4. Results

### 4.1. Screening of the Nitrile-Degrading Bacteria

**Figure 1:**
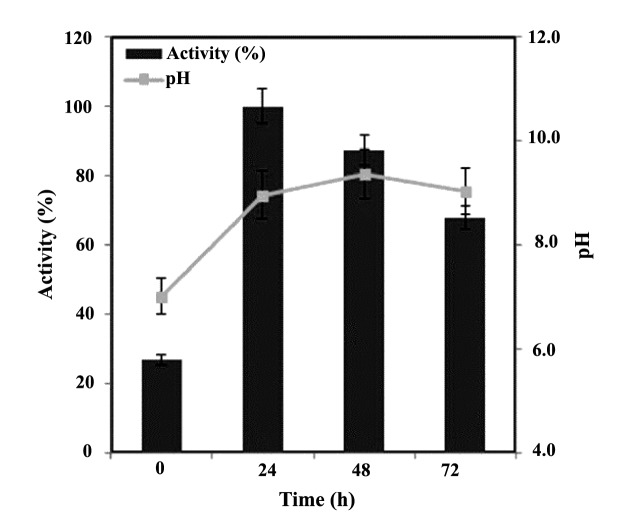
pH of the growth medium and concentration of ammonia liberated from acetonitrile degradation. *Pseudomonas aeruginosa* RZ44 was cultured and samples picked up at 24 h intervals. All experimental data are means ± standard deviation (SD) of triplicate determinations

Water/soil samples were collected from the sewage sites in the Kerman city (southeastern, Iran), and were stored at -20°C. The primary isolations was done byinoculation of these samples on the minimal nutrient media (MM1) containing 1% acetonitrile as the solecarbon source. After incubation at 30°C for 5 days, samples were picked up and inoculated into flasks containing fresh medium. Subsequently, flasks were incubated for 3 days at 30°C. After that, the samples were picked up and streaked on the minimal nutrient plates containing phenol red as a pH indicator. As well, plateswere overlaid with 100 μL acetonitrile before streakingand then incubated in the 30°C for 3 days. 13 isolates were examined for their ability to change the color of medium from yellow to pink, which was an indicator of the ammonia production from acetonitrile. A single strain (designated as RZ44) which showed a higher pink halo around the bacterial growth zone in the minimal nutrient medium plate compared to the other isolates was selected for further characterization. The pH of the medium and production of ammonia by acetonitrile are shown in (Figure 1). These results indicate that degradation of acetonitrile by *P. aeruginosa* strain RZ44 increases the pH of the growth medium from initial pH 7.0 to 9.37.

### 4.2. Biochemical and Molecular Characterization

**Figure 2:**
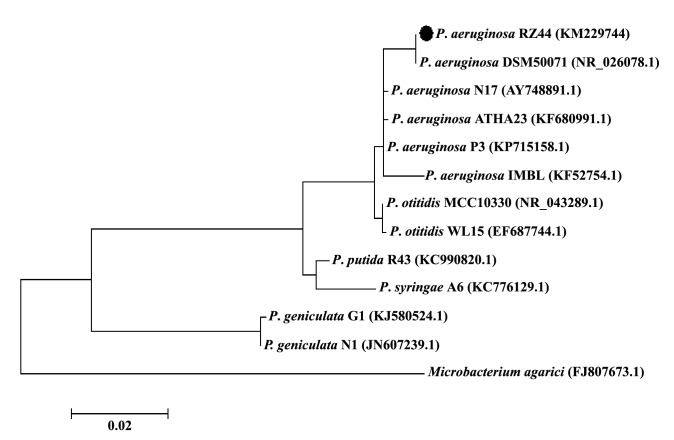
The phylogenetic dendrogram constructed for *Pseudomonas aeruginosa* RZ44 and related strains based on the 16S rRNA sequence. Microbacterium agarici was used as a outgroup

The physiological characteristics of the RZ44 strain were also investigated. Strain RZ44 was a Gram negative, non-spore forming bacterium. Catalase and oxidase reactions were positive, as well as liquefaction of gelatin, nitrate reduction, in addition to hydrolysis of the casein. Starch hydrolysis, Voges-Proskauer test, methyl red test, urease, indole and H2S production were negative (40). These data indicated that strain RZ44 resembles to a member of *Pseudomonas* genus. Strain RZ44 was 100% identical in the sequence with *Pseudomonas aeruginosa* by examining phylogenetic tree (Figure 2) according to the 16S rRNA sequence. The sequence was deposited in the GenBank database with accession no. KM229744.

### 4.3. Investigation of the Medium Optimization

**Figure 3:**
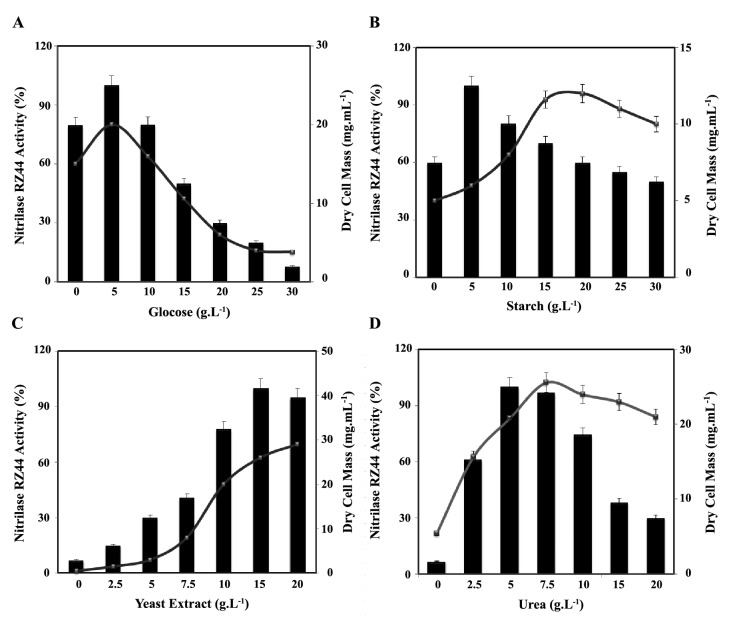
Effects of different concentratios of the A: glucose, B: starch, C: yeast extract, and D: urea on the bacterial growth and nitrilase production of *P. aeruginosa* RZ44. Enzyme production was measeured after 48 h incubation of *P. aeruginosa* RZ44. All experimental data are means ± standard deviation (SD) of triplicate determinations

The strain RZ44 was grown in different carbon sources (10 g.L^-1^) in order to observe the activity pattern of the nitrilase. As the results shown in (Table 1), glycerol slightly decreased nitrilase production, while glucose and starch strongly supported the nitrilase production, compared to the control. Furthermore, in the present study, we have also investigated the effect of different concentration of glucose and starch to find the best concentration of these components for nitrilase and biomass production. In these experiments, other media components and physiological parameters were kept constant, and the activity was analyzed after 48 h of growth. The results in (Figure 3) indicate that 5 g.L^-1^ glucose and starch is the optimal concentration of the nitrilase production.

**Table 1:** The effect of different carbon and ion sources on the bacterial growth and nitrilase production of *P. aeruginosa* RZ44

**Carbon source**	**Activity (%)**	**Biomass (mg.ml** ^-1^ **)**	**Compounds**	**Activity (%)**	**Biomass** **(mg.ml** ^-1^ **)**
Control	100	9.6	Control	100	14.0
Glucose	820±2.1	80.0	ZnSO_4_	94.0±1.2	12.0
Fructose	159±3.2	15.0	MnSO_4_	82.0±1.3	8.0
Manitol	180±2.1	5.0	MgSO_4_	142.0±1.3	30.0
Maltose	404±1.6	22.0	FeSO_4_	130±1.2	35.0
Sucrose	123±1.6	20.0	CuSO_4_	70±2.2	9.30
Starch	550±1.3	22.0	CaCl_2_	87±2.1	14.8
Acetate	225±2.2	21.0	CoCl_2_	80±1.6	16.4
Citrate	360±3.1	22.0	NaCl	170±2.6	40.0
Glycerol	73±2.1	8.2			

As one of the major components of the culture medium, the effects of various nitrogen sources (5 g.L^-1^) was also investigated in this study (Table 2). As shown in Table 2, the lowest biomass and nitrilase production could be detected when sodium nitrate and Triton X^-1^00 are used as the nitrogen sources, in contrast, urea and yeast extract favor the more biomass and nitrilase production. The mentioned factors were selected as the suitable nitrogen sources for further study. Results in figure 3 show that the 15 g.L^-1^ yeast extract and 5 g.L^-1^ urea are the optimum concentration for the nitrilase production.

**Table 2:** The effect of different nitrogen sources and different pHs on the bacterial growth and nitrilase production of *P. aeruginosa* RZ44

**Nitrogen sources**	**Activity (%)**	** Biomass (mg.mL^-1^) **	**pH**	**Activity (%)**	** Biomass (mg.mL^-1^) **
Control	100	5.7	5.0	80.0±2.5	52
Yeast extract	650±2.1	7.0	6.0	92.0±2.1	60
pepton	400±3.2	4.1	7.0	100±1.9	65
T X100	140±2.1	4.3	8.0	90±1.8	60
Urea	780±1.6	11.3			
NaNO_3_	120±1.7	8.4			

The effect of the pH on *P. aeruginosa* RZ44 nitrilase production was examined by changing the pH of the production medium in a range from 5.0 to 8.0 and allowing the culture to grow for 48 h at 30oC. The speed of the shaker was adjusted to168 rpm. Nitrilase activity at the different pH of the medium was assessed and are summarized in Table 2. These results show that nitrilase activity increases in a range of pH from 5.0 to 7.0; then it starts to decrease. The optimum pH for nitrilase production is 7.0, therefore, in subsequent studies, the pH of the medium was adjusted to 7.0 before sterilization.

In this study, the effects of various metal ions (0.2 g.L^-1^) on biomass and nitrilase production was also investigated. The medium without metal ion was considered as the control, while the other ingredients were kept unchanged. As shown in Table 1, the addition of Mg^2+^, Fe^2+^, and Na^+^ support the biomass and nitrilaseproduction.

### 4.4. Enzyme Characterization

**Figure 4:**
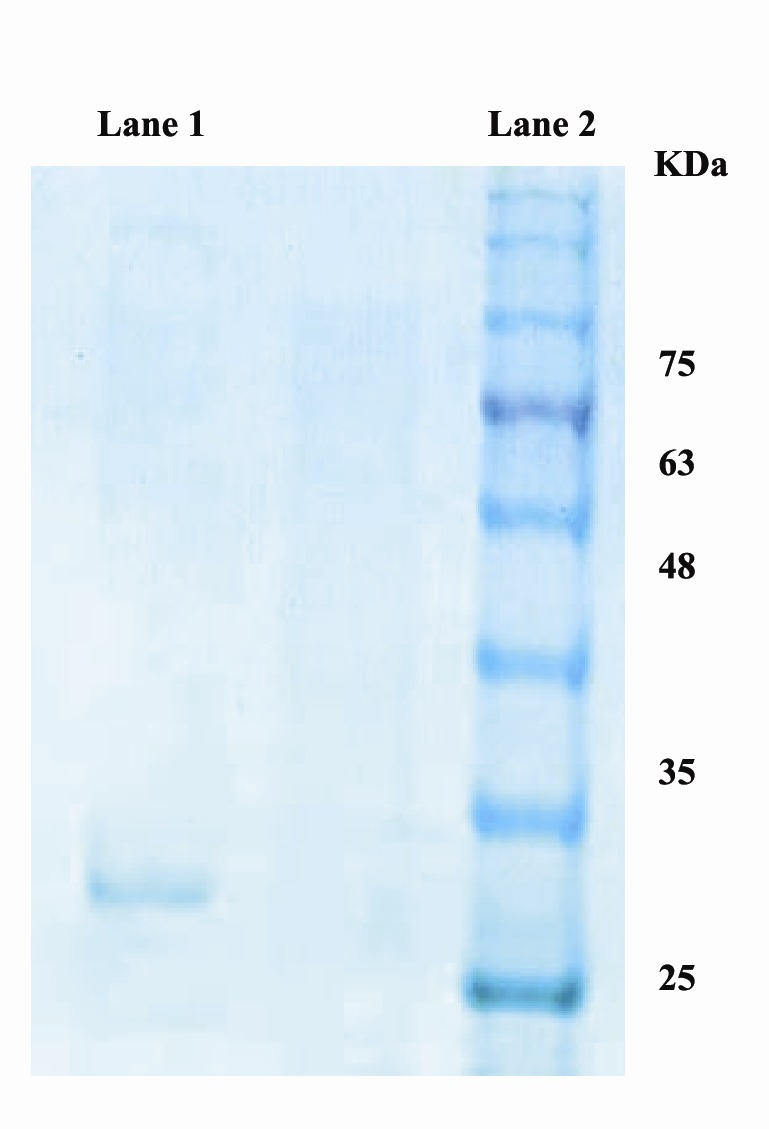
SDS-PAGE assessment of the purified nitrilase RZ44 (Lane 1) and the protein molecular weight markers (Lane 2)

The apparent molecular mass of the purified RZ44 nitrilase as obtained by SDS-PAGE is about 30 kDa (Figure 4). The molecular weights of nitrilase from other bacterial species have been reported as 45 kDa for *Bacillus cereus* strain FA12 (41), 43 kDa for *Pseudomonas putida* (42), and 33 kDa for *P. fluorescens* Pf5 (23).

#### 4.4.1. Effect of pH on the Enzyme Activity and Stability

**Figure 5:**
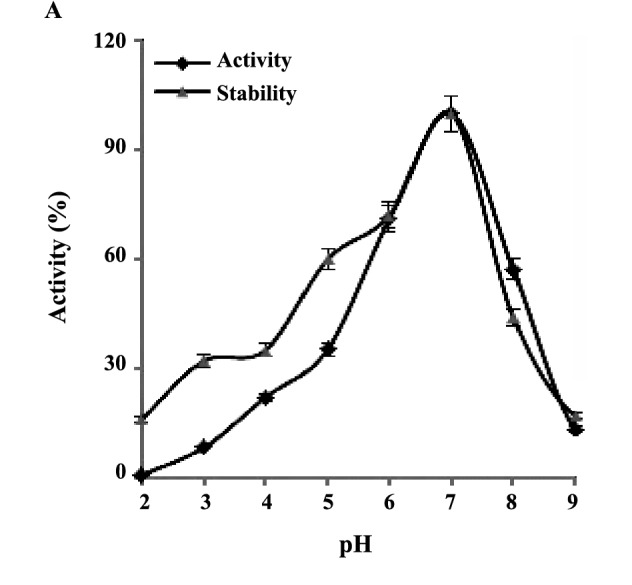
The effect of A: pHs (2.0-9.0) and B: temperatures (T) (30-90°C) on the relative nitrilase activity and stability. Enzyme activity was evaluated by determining the production of ammonia following to the modification of the phenol/hypochlorite method as described in the material and methods section. All experimental data are means ± standard deviation (SD) of triplicate determinations

To investigate the effect of pH on the enzyme activity and stability, buffers including Na-acetate (pH 4.0-6.0), phosphate (pH 6.0-8.0), and Tris-HCl (pH 7.0-10.0), with a concentrations of 50 mM for each were used. According to the standard method described above, the relative residual activity was measured for the pH stability assessment. The enzyme shows the highest activity and stability comparatively at pH 7.0, as it drastically is lost in the low and the high pH. The enzyme retained more than 50% activity at pH 6.0 and 8.0 (Figure 5).

#### 4.4.2. Effect of Temperature on the Enzyme Activity and Stability

Since the temperature is the key factor for reaction rate, the effect of temperature on the nitrilase activity was investigated at various temperatures (30-90oC). The results showed that the optimum temperature for the nitrilase activity and stability is 40oC, which then gradually subjects to the reduction. Enzyme activity was about 60% at 60°C (Figure 5). It is well known that thermostability is an index of a significant importance for the nitrilase. In this study, the thermostability of the nitrilase was found to be comparatively acceptable under 60oC. According to the figure 5, thermostability of the RZ44 nitrilase was about 85% of the initial thermostability at 60oC.

#### 4.4.3. Effect of Metal Ions and Chemicals on Enzyme Activity

The effects of various metal ions and reagents on the enzyme activity was investigated. The enzyme was assayed under standard condition with 1 mM concentrations of the various reagents. RZ44 nitrilase was entirely repressed by Co^+2^ and Ca^+2^, while it was inhibited almost 60% in the presence of MnSO_4_ and CuSO_4_. The enzyme activity was slightly increased in the presence of Fe^+2^ and Mg^+2^ (Table 3).

**Table 3:** The effect of different carbon and ion sources on the bacterial growth and nitrilase production of *P. aerugi- nosa* RZ44

**Compounds (1 mM)**	**Activity (%)**
No compound	100
FeSO_4_	107±2.1
MgSO_4_	110±3.2
ZnSO_4_	80±2.1
MnSO_4_	41±1.6
CuSO_4_	39±1.6
ZnCl_2_	76±1.3
CoCl_2_	16±0.2
CaCl_2_	12±0.1
FeCl_3_	52±2.2
DTT	85±0.3
EDTA	93±1.2
H_2_O_2_	27±1.7

#### 4.4.4. Substrate Specificity and Biotransformation

The Michaelis-Menten constant (*K*m) of the nitrilase RZ44 for acetonitrile and acetamide was determined by varying the substrate concentration from 0.05 to 30 mM and calculated by Lineweaver-Burk equation. The substrate

activity of the nitrilase RZ44 toward diverse nitriles with different structural specificity was considered. The relative activities for different nitrile substrates were determined by measuring the extent of ammonia released through the hydrolysis; the results of which are listed in Table 4. Nitrilase RZ44 had very broad substrate specificity, hydrolyzing aliphatic, aromatic, and heterocyclic nitriles. There was a slight preference of the nitrilase RZ44 for aliphatic nitrile compounds as substrate (entries 1, 4, 5). A lower activity was also detected against aromatic nitriles (entries 2, 4, 11, 12). Amide substrates (6-8,10,15) were also effectively metabolized by nitrilase RZ44. The maximum activity was detected against benzamide with almost 2.24 folds of enzymatic activity, but, nitrilase RZ44 showed a lower substrate hydrolysis activity in the presence of urea and glutamine (Table 4).

**Figure 6:**
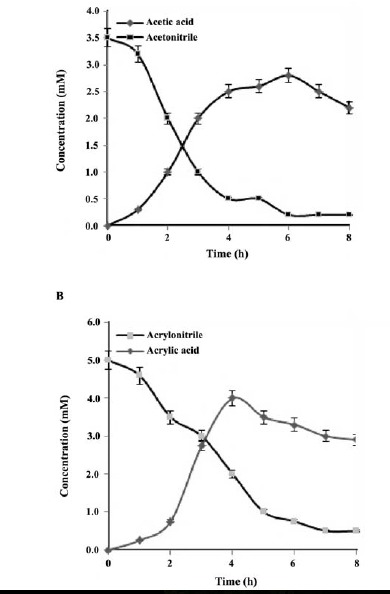
Conversion time on the biotransformation of the nitrile substrates. A: acetonitrile and B: acrylonitrile to the corresponding acid (acetic acid and acrylic acid), by P. aeruginosa RZ44. All experimental data are means ± standard deviation (SD) of triplicate determinations

Biotransformation of the several nitriles by *P. aeruginosa* RZ44 resting cells is shown in figure 6. These data were obtained by GC chromatography as described in the material and methods. The conversion data of the acetonitrile (3.5 mM) and acrylonitrile (5 mM) into the corresponding acid products by the *P. aeruginosa* RZ44 strain are shown in (Figure 6). Time course analysis of the nitrile conversion by the resting *P. aeruginosa* RZ44 cells showed that substrates (*i*.*e*. nitrile) was hydrolyzed within 8 h.

**Table 4:** Substrate specificities of the RZ44 nitrilase. Nitrilase activity was measured in the presence of each sub- strate

**Substrate**	**Activity (%)**
Acetonitrile	100
Benzonitrile	94±0.2
2-amino-benzonitrile	88±1.1
Acrylonitrile	107±0.8
Malononitrile	44±0.8
Acetamide	119±0.5
Acrylamide	101±0.2
m-methyl-propionamide	161± 0.5
Bis acrylamide	67±0.4
Benzamide	257±0.8
pyridine	23±0.7
3-Cyanopyridine	102± 0.8

## 5. Discussion

In this study, a nitrilase producing *Pseudomonas aeruginosa* was isolated and identified, which has potential for nitrile biotransformation. This strain produces a pink halo zone in the medium supplemented with 1% acetonitrile and phenol red.

From the perspective of the practical industrial application, the growth media strategy and reaction constraints are critical and need to be developed for the highest conversion of the nitriles (34). Consequently, we have examined the optimization of the different cultures condition that play a significant role in the enzyme production from *P. aeruginosa* RZ44. Results have indicated that glycerol has slightly decreased nitrilase production, while glucose and starch have strongly supported nitrilase production. Furthermore, results have shown that 5 g.L^-1^ of glucose and starch are the optimal concentration for nitrilase production. Our results agrees with what obtained for *Rh. erythropolis* ZJB-0910, for which sucrose induces lower nitrilase production than other carbon sources such as starch and glucose (33). But, it does not in agree with the *Streptomyces* sp. MTCC 7546. In spite of that, the results obtained through working on the culture medium that was carried out on *Streptomyces* sp. MTCC 7546 show that nitrilase production is not specific to a single carbon source (32). Our results also agree with the results reported by Zheng *et al*. that have shown 20 g.L^-1^ glucose does properly affect the biomass and nitrilase production (37). In addition, Nageshwar and co-workers have also reported that *A. faecalis* MTCC 10757 nitrilase activity was maximal, when glucose was used as the carbon source. They have shown that 10 g.L^-1^ is the optimal glucose concentration (34).

Results also have shown that 15 g.L^-1^ yeast extract and 5 g.L^-1^ urea are the optimum concentration of these two medium constituents for nitrilase production. Dong *et al*. have also reported that yeast extract and peptone are the preeminent nitrogen sources for nitrilase production by *Rh. erythropolis* ZJB-0910 (33). They have also reported that biomass production by ZJB-0910 strain reduces with yeast extract at a concentration above 8 g.L^-1^, in which nitrilase activity slightly improves (33).

The optimum pH for the production of nitrilase is 7.0 and therefore in subsequent studies the pH of the medium were adjusted to the 7.0 before sterilization. The nitrilase-producing media are recognized to favor enzyme synthesis mostly at the neutral pH (23,34,43). The optimal pH for nitrilase production was 7.0 by *Streptomyces* sp. MTCC 7546 (32). Nageshwar and co-workers have reported that the most promising pH for nitrilase production is between 7.0 and 7.5 by *A. faecalis* MTCC 10757 (34).

It was also shown that addition of Mg^2+^, Fe^2+^, and Na+ supports the biomass in addition to nitrilase production. Co^2+^, Mn2+ and Cu^2+^ were confirmed to inhibit cell growth and enzyme activity. These results are in agreement with the results obtained for *Rh. Erythropolis* ZJB-0910. Nitrilase production by *Rh. Erythropolis* ZJB-0910 was slightly improved by Mg^2+^ and inhibited by Ca^2+^, Cu^2+^, and Zn^2+^ (33).

Results obtained on nitrilase characterization has also shown that, the enzyme exhibits high activity and stability comparatively at pH 7.0, as its activity was drastically lost in the low and high pH. According to these results, phosphate with pH 7.0 was selected as the optimum buffer for the biotransformation experiments. The nitrilase-producing bacteria showed the best nitrilase activity in the pH range 7.0-8.0 (23,34). It is well known that the thermostability is an index of significant importance for nitrilase. The results obtained with respect to enzyme thermostabality have shown that the optimum temperature for the nitrilase activity and stability is 40oC, as it gradually reduced. The optimal temperature of the nitrilase activity of *Mesorhizobium* sp. F28 was found to be around 37-45°C, but this enzyme was inactive at 55-65°C (44). Nageshwar and co-workers have reported that the optimal temperature for maximum activity would be between 20 and 30°C and the nitrilase activity was found to be maximum at 35°C by *A. faecalis* MTCC 10757 (34).

As well, RZ44 nitrilase was entirely repressed with Co^+2^, Ca^+2^, while the inhibition in the presence of MnSO_4_ and CuSO_4_ was about 60%. This enzyme activity was slightly increased in the presence of Fe^+2^ and Mg^+2^. *Nocardia* sp. NCIB 11216 nitrilase was also repressed by Fe^+3^ and Mn^+2^. Hydrogen peroxide reduced the nitrilase activity of RZ44 by about 70%. Feng and coworkers reported that, *Mesorhizobium* sp. F28 was sensitive to CuSO4, Ag_2_SO_3_, which are sulfhydryl containing salts. This research has implied that the nitrilase active site of the *Rh. erythropolis* N04 is consisted of cysteine and serine, since the nitrilase was repressed to a significant degree by several sulfhydryl and oxidizing elements. Consequently, the nitrilase active site of these strains might contain cysteine and serine residues (44).

The chelating agent such as EDTA had diverse effects on the nitrilase activity of the bacteria. EDTA was shown to have no major effect on the activity of *P*.*aeruginosa* RZ44 nitrilase. This finding is in agreement with the nitrilase of *R. rhodochrous* J1 and *B. pallidus* Dac521, which are unaffected by 1 mM and 10 mM EDTA, respectively (17,19). *Mesorhizobium* sp. F28 nitrlase was diminished about 64% by EDTA (44).

Additionally, the reducing agents such as beta-mercaptoethanol and DTT have no major effect on the nitrilase activity of *P. aeruginosa* RZ44, as observed in *R. rhodochrous* J1 (17), *B. smithii* SC-J05-1 (45), *B. pallidus* Dac521 (19) and *Mesorhizobium* sp. F28 (44). *K*m value has indicated that acetonitrile was noticeably the preferred substrate for RZ44 nitrilase when compared with the acetamide. But, the specific activity of RZ44 nitrilase for acetamide was 2 times more than that of acetonitrile. The specific activity of RZ44 nitrilase was about 616 and 1383 U.mg^-1^ for acetonitrile and acetamide, respectively. The specific activities for *R. rhodochrous* J1 (115 U.mg^-1^ dcw) (17), *R. pyridinovorans* MW3 (97 U.mg^-1^ dcw) (46), *B. imperialis* CBS 498-74 (47 U.mg^-1^ dcw) (47), and *R. rhodochrous* PA-34 (55.0 U.mg^-1^ dcw) (25) have been reported. These results indicated that RZ44 has high substrate tolerance and higher nitrilase activity.

RZ44 nitrilase shows a broad substrate specificity, hydrolyzing the aliphatic, aromatic, and heterocyclic nitriles. However, it was found a slight preference for RZ44 nitrilase toward aliphatic as nitriles as substrate (entries 1, 4, 5). Alower RZ44 nitrilase activity was also detected toward aromatic nitriles (entries 2, 4, 11, 12). Amide substrates (6-8,10,15) were also effectively metabolized by RZ44 nitrilase. The maximum activity was detected with benzamide about 2.24 folds, but, RZ44 nitrilase show lower substrate hydrolyzing activity in the presence of urea and glutamine. Similarly, the nitrilases produced by *Bacillus pallidus* (19), *R. rhodochrous* K22 (43), *Bacillus subtilis* ZJB-063 (37),

*Nocardia globerula* NHN-2 (20) and *Amycolatopsis* sp. IITR215 (48), show a broad substrates, as they hydrolyze aliphatic, aromatic and heterocyclic nitriles.

Biotransformation assessment by GC chromatography also confirms that the *P. aeruginosa* RZ44 resting cells directly hydrolyze nitrile substrate to the corresponding acid, similar to that of *Rh. rhodochrous* K22 (43) and *A. faecalis* (IICT-A3) (34).

It was also revealed that the quantity of acrylic acid and acetic acid identified from the conversion of the correspond nitrile is maximum after 4 and 6 hours of incubation with acrylonitrile and acetonitrile, respectively. The time course of acetonitrile (3.5 mM) and acrylonitrile (5 mM) conversion into their corresponding acids by *B. cereus* FA12 resting cells showed that these nitriles were hydrolyzed within 12 h, and the amount of ammonia produced from acetamide and acetonitrile was maximum after 5 and 7 hours of the incubation, respectively (41). Remarkably, the quantity of the acetic and acrylic acids identified from the conversion of acetonitrile and acrylonitrile showed a small aberration from the theoretical quantity which was equivalent to the quantity of acetonitrile and acrylonitrile added (3.5 and 5 mM, respectively). This observation has exhibited that the amide or acid was partially converted by *P. aeruginosa* RZ44. In addition, an increased quantity of acid was used with the increase in conversion time.

## 6. Conclusions

*P. aeruginosa* RZ44 is a strain which was isolated from the sewage of the city of Kerman. *P. aeruginosa* RZ44 nitrilase shows broad substrate specificity for hydrolysis of the aliphatic, aromatic, and heterocyclic nitriles. Medium optimization showed that some environmental conditions play significant role in the nitrilase production from *P. aeruginosa* strain RZ44. The maximum nitrilase production was detected in the presence of glucose, yeast extract, and Mg^+2^ following to 24 h of incubation. It was mentioned that, using resting cells as catalyst, there is no need for an additional immobilization of the catalyst before use, and it would diminish the cost of production to a large amount and saving of the time, as well. Results have indicated that *P. aeruginosa* strain RZ44 has an excellent nitrile conversion capacity, which can be used for nitrile biotransformation.

## Acknowledgements

The authors express their gratitude to the Research Council of the Shahid Bahonar University of Kerman‏ and Iran National Science Foundation (INSF) for their‏ financial support during the course of this project.
